# Molecular Characteristics and Processing Technologies of Dairy Products from Non-Traditional Species

**DOI:** 10.3390/molecules29225427

**Published:** 2024-11-18

**Authors:** Isabela Pérez Núñez, Rommy Díaz, John Quiñones, Ailín Martínez, Lidiana Velázquez, Rodrigo Huaiquipán, Daniela Tapia, Alex Muñoz, Marcos Valdés, Néstor Sepúlveda, Erwin Paz

**Affiliations:** 1 Doctoral Program in Agrifood and Environment Sciences, Universidad de La Frontera, Temuco 4780000, Chile; i.perez04@ufromail.cl (I.P.N.); r.huaiquipan01@ufromail.cl (R.H.); d.tapia01@ufromail.cl (D.T.); a.munoz45@ufromail.cl (A.M.); m.valdes09@ufromail.cl (M.V.); 2 Meat Quality Innovation and Technology Centre (CTI-Carne), Universidad de La Frontera, Temuco 4780000, Chile; john.quinones@ufrontera.cl (J.Q.); a.martinez26@ufromail.cl (A.M.); lidivm15@gmail.com (L.V.); nestor.sepulveda@ufrontera.cl (N.S.); 3 Faculty of Agricultural and Environmental Sciences, Universidad de La Frontera, Av. Francisco Salazar 01145, Temuco 4811230, Chile; 4 Doctoral Program in Science Major in Applied Cellular and Molecular Biology, Universidad de La Frontera, Av. Francisco Salazar 01145, Temuco 4811230, Chile; 5 UWA Institute of Agriculture, The University of Western Australia, Perth 6009, Australia; erwin.pazmunoz@uwa.edu.au

**Keywords:** milk, non-traditional animals, molecular characteristics, processing technologies

## Abstract

Non-bovine dairy animals, commonly referred to as non-traditional dairy species, include goats, sheep, yaks, buffalo, donkeys, alpacas, llamas, and other less commonly farmed species. These animals have been integral to livestock systems since ancient times, providing milk and other essential products. Despite their historical significance, dairy production from many of these species remains predominantly confined to rural areas in developing countries, where scientific advancements and technical improvements are often limited. As a consequence of this, the scientific literature and technological developments in the processing and characterization of dairy products from these species have lagged behind those for cow’s milk. This review aims to compile and analyze existing research on dairy products derived from non-traditional animals, focusing on their molecular characteristics, including proteins (alpha, beta, kappa, and total casein), fats (cholesterol and total fat), lactose, albumin, ash, total solids, and somatic cell count, among others, for each of these species. Additionally, we discuss emerging technologies employed in their processing, encompassing both non-thermal methods (such as high-pressure processing, pulsed electric fields, ultrasound processing, UV-C irradiation, gamma radiation, microfiltration, and cold plasma processing) and thermal methods (such as ohmic heating). This review also explores the specific potential applications and challenges of implementing these technologies. By synthesizing recent findings, we aim to stimulate further research into innovative technologies and strategies that can enhance the quality and yield of non-bovine dairy products. Understanding the unique properties of milk from these species may lead to new opportunities for product development, improved processing methods, and increased commercialization in both developing and developed markets.

## 1. Introduction

Non-bovine dairy animals, also known as non-traditional dairy animals, include species such as goats, sheep, yaks, buffalo, donkeys, alpacas, llamas, and others. The use of these animals as livestock dates back to ancient times, where they were utilized by various cultures to provide food and clothing for the human groups that maintained them.

Dairy animals play a fundamental role in social, cultural, and economic development in many developing countries, particularly in areas of Asia and Africa, where they provide the population with milk, meat, wool, leather, and other products [1]. Currently, in areas with difficult access to bovine milk products, such as arid and semi-arid regions, goats and sheep play a crucial role in food security and poverty alleviation [2,3].

The global goat population has shown a significant increase in recent decades, growing 52% worldwide and 56% in developing countries between 1981 and 2001 [4]. By 2011, the global goat population exceeded 875.5 million, with Asia and Africa accounting for 93.2% of the total population [5]. By 2019, it exceeded one billion animals [6], and in India, which holds the largest goat population in the world, there were 148.88 million goats as of the latest available data [7]. The Indian goat population is predicted to increase to 162.32 million by 2031 [7]. This tendency is also observed in Ethiopia, where the combined bovine, ovine and caprine cattle population grew from 54.5 million to 103.5 million between 1995 and 2013 [8].

The ovine milk industry has also grown both globally and in specific countries. In the United Stated, commercial sheep milking began around 30 years ago and continues to expand, with potential for additional growth, as suggested by the increase in cheese imports [9]. In New Zealand, the commercial goat-milking industry is emerging, supported by current investigations in milk production, environmental impact, and milk composition [10]. Small ruminants, particularly in Asia, exhibit significant diversity and wide distribution. The sheep-milk industry holds substantial economic potential due to its high nutritional value, derived products, and the genetic factors that influence its production and composition [11], with growing global demand for these products [1]. In addition to goats and sheep, other dairy-producing species, such as camels, buffaloes, equines, and yaks, are commonly raised in developing countries or areas with strong ancestral/cultural influences. These animals are recognized as producers of both milk and highly nutritious meat, which is internationally regarded as highly innovative [12]. This potential and cultural relevance have increased the need for scientific, educational, and functional infrastructure to make these products; however, there are limited indicators of the global population and production status of these less common animals, with most indicators being calculated by countries independently. According to the Food and Agriculture Organization (FAO), “Cattle produce 81% of world milk production, followed by buffaloes with 15%, goats with 2% and sheep with 1%; camels provide 0.4%. The remaining share is produced by other dairy species such as equines and yaks” [13]. Approximately one-third of milk production in developing countries is derived from buffaloes, goats, camels, and sheep, whereas in developed countries, nearly all milk production is from cattle. Non-cattle dairy species contribute 38% of milk production in Asia, 22% in Africa, 3% in Europe, and 0.5% in the Americas, while their contribution is minimal in Oceania [13]. Previous studies have explored the potential of these products [14]; however, this work aims to include some of the less common alternatives, examine existing advancements, and propose further improvements to advance this industry.

## 2. Current Context of the Non-Traditional Dairy Animal Industry

### 2.1. The Global Dairy Industry of Non-Traditional Animals

The goat and sheep dairy industry has considerably grown in recent decades. Worldwide, the dairy-goat population grew 52% between 1981 and 2001, while dairy sheep increased by 14% in developing countries by 2001 [4]. Despite the great number of animals, sheep and goat dairy represented only 1.3% and 1.9%, respectively, of the global milk production in 2016 (799 million tons) [15]. The industry’s growth has been influenced by increased recognition of non-bovine products, its relation to specific territories, and the generation of new dairy production ecosystems [16]. The greater capability of these species to adapt to less favorable conditions [17,18,19] and to provide other services and products such as meat, manure, and work power have driven further recognition of these animals’ relevance for the current dairy industry [20,21,22].

Goats are efficient converters of low-quality forage into meat and milk due to their unique physiological and behavioral adaptations. They exhibit selective feeding behavior, allowing them to consume higher-quality rations when provided with excess feed [23]. This ability, along with their resilience and adaptability, makes them suitable for various production systems [24]. Additionally, they emit less methane than other ruminants, such as cattle [25,26], which has led to proposals that the growth of this industry and its potential to supplement the beef and dairy markets could positively contribute to climate change adaptation [25]. Sheep and goat milk possess characteristics that classify them as high-quality milk, particularly in the case of European dairy breeds, making their products eligible for classification as “gourmet” [27].

Dairy-production animals such as sheep, goats, and buffalo play a crucial role in the economy of developing countries by providing food security, employment, and income generation [6,28]. Challenges include low economic returns, the use of outdated technologies, and high production costs. Studying the genetic potential and local diversity is necessary to increase milk production in accordance with regional conditions. In the Americas, goat-milk production faces issues such as production efficiency, waste management, and animal welfare [28].

### 2.2. Non-Traditional Animals in the Dairy Industry

**Figure 1:**
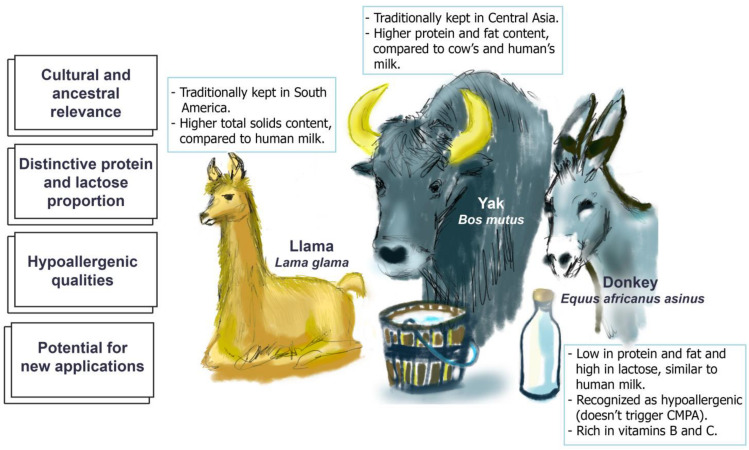
Main benefits of non-traditional animals’ milk.

In recent decades, there has been significant interest in developing industries related to non-traditional dairy animals. Historically, these animals were integral to ancient civilizations, providing economic stability and nutritional resources. As research into these species has expanded, their unique traits have been increasingly elucidated. Their products are recognized for being highly nutritious, hypoallergenic, and suitable for producing specialty items such as cheeses, personal-care products, and infant formulas. The key distinguishing features of milk from alternative dairy animals, compared to cow’s milk, are illustrated in Figure 1. The following section will provide a comprehensive overview of the currently available information regarding some of the most commonly utilized non-traditional dairy animals.

#### 2.2.1. Goats

Goat population has increased considerably in recent times [5,6,7], as has caprine production. In a review by Pardo et al., it is mentioned that “highly selected caprine farming has increased in popularity” during the present decade [24]. This trend is attributed to recent advances in genetics that have improved goat selection efficiency, and by the search for alternatives to traditional cow’s milk, considering goat milk’s nutritional characteristics [29,30], hypoallergenicity [31], and ease of digestion [32]. Additionally, goat milk has potential for producing specialty products such as infant formula [29], personal-care and hygiene items [33], and gourmet cheeses [34].

In some developed countries, mainly in Europe, goat dairy production is carried out using specialized milking machines, partly owed to states’ inversion and governmental support [6]. In developing countries, the lack of productive organization, marketing strategies, and government interest present challenges that need to be overcome [35,36]. According to the Food and Agriculture Organization of the United Nations (FAO), global milk production reached 19.4 million metric tons in 2017 [37], representing an increase of 16% from 2007 to 2017 [38]. Goat-milk production has increased by 22% in Asia, 13% in Africa, 9% in Oceania, 5% in the Americas, and 4% in Europe [6].

Some of the challenges faced by goat production in some countries are modernization and product safety. In many cases, production is local and family-based, with production techniques being carried out in a rustic manner, often lacking necessary sanitary precautions. Schmidt (2021) suggests that in Rio Grande do Sul, goats are kept under suboptimal production conditions [39]. Sánchez-Macías et al. (2013) discuss the impact of somatic cell count (SCC) on the quality of fresh goat-milk cheese, implying the need of a level of quality control in the production process [40]. Raynal-Ljutovac et al. (2007) emphasize the importance of SCC as a measure of hygienic quality in milk, advocating for mastitis-control programs to improve sanitary conditions [41]. Ahmed et al. (2022) highlight unsanitary milk-handling practices in Ethiopia, similar to some traditional practices in India, suggesting a common challenge in improving sanitary conditions in developing regions [42]. There is evidence of regulated and hygienic practices in goat-milk production and its products [40,41,43]; therefore, it is inaccurate to generalize that goat milk is produced under unsanitary and outdated conditions worldwide. Technological and sanitary conditions are evolving, with a recognized need for infrastructure development, improvement in feeding practices, and political support to fully realize the sector’s potential [44]. Implementing proper hygiene practices is crucial for enhancing milk quality and safety. Studies have shown that many farmers lack essential sanitary procedures. For instance, in one study, 94.8% of farmers did not eliminate the first jets of milk, only 29.2% used a screened mug test for mastitis detection, and just 41% performed pre- and post-dipping [45]. Educating farmers on proper cleaning techniques, udder hygiene, and milk handling is essential to reduce contamination risks. Technological innovations can significantly improve working conditions and attract younger generations to goat farming. Strategies like processing more milk into cheese or other dairy products in production areas and promoting technological advancements can make the sector more appealing and efficient [3,46]. Additionally, adopting clean, green, and ethical practices for responsible innovation in developing countries can optimize natural and local resources, transitioning from risk reduction to increased productivity [3].

Proteomic assays have demonstrated a high and complex variety of proteins depending on breed, lactation stage, and processing method applied to the product. Technological advances, such as two-dimensional differential gel electrophoresis (2D-DIGE), have facilitated the characterization of the proteomic profile in the Girgentana breed, providing a reference map that includes proteins such as milk fat globule EGF factor, β-lactoglobulin, β-casein, and serum albumin [47].

It has been shown that proteolytic conditions and protein composition influence the hydrolysis of goat milk proteins, where caseins are rapidly hydrolyzed, and low-abundance immunoactive proteins exhibit greater resistance. This suggests a balance between protein utilization and immune protection [48].

Proteomic analyses have also identified variations in whey-protein composition throughout the lactation cycle. A difference in the abundance of 238 proteins was observed over the course of lactation. Some of the differentially expressed proteins include α-2-macroglobulin, immunoglobulin, peptidoglycan-recognition protein, LBP, lactoferrin, lactoperoxidase, and melanotransferrin, which are related to immune function; complement C3 and C5, complement subcomponent C1r, and fibronectin, which decrease between days 1 and 240 and are associated with coagulation cascades, essential for the immune response; α-1-antiproteinase, inter-α-trypsin inhibitor, plasminogen, and antithrombin-III, which decrease between days 1 and 240 and are linked to protease activity, maintaining milk stability and preventing the degradation of immune-related proteins; and FABP3, nucleobindin 2, calgranulin A, and calgranulin B, which increase from day 1 to day 240 and are related to nutrient transport [49]. These characteristics could be beneficial for the implementation of goat milk to human diets, especially for those with dietary requirements, such as infants and the elderly. Comparative studies of goat breeds have shown specific differences between protein profiles, which could influence the nutritional and biologically active properties of the milk. Nasalean et al. (2017) compared the protein profiles of French Alpine and Romanian Carpathian goats using SDS-PAGE. Differences were identified in the expression of immunoglobulin (4% in Alpine; 2.7% in Carpathian), β-casein (26% in Alpine; 30.56% in Carpathian), κ-casein (12.3% in Alpine; 15.4% in Carpathian), and β-lactoglobulin (23.85% in Alpine; 17.9% in Carpathian) [50]. These studies contribute to a deeper understanding of the nutritional and functional properties of goat milk, with potential applications in human nutrition and health.

Additionally, the response of goat-milk proteins during endotoxin-induced mastitis has been studied [51], where a sustained presence of casein proteins and the emergence of haptoglobin, serum amyloid A, lactoferrin, and cathelicidins were observed, indicative of an inflammatory response. An increase in somatic cell count was also noted following exposure to bacterial LPS for the induction of experimental mastitis. Misri et al. reported an increase in total proteins, including immunoglobulins, in milk during acholeplasmic mastitis, suggesting a humoral immune response [52]. Pisanu et al. identified differential proteins in milk infected with *Staphylococcus aureus*, including haptoglobin and cytoskeletal proteins, which are associated with defense and inflammatory processes [53]. These proteins may serve as more reliable markers of intramammary infections (IMIs) than the somatic cell count (SCC), especially in the late stage of lactation.

Interestingly, Pisanu et al. also noted an unexpected increase in serum amyloid A in uninfected milk during the late lactation phase, which is typically associated with inflammation. This highlights the complexity of the proteomic response to mastitis and the potential influence of physiological factors, such as lactation stage, on protein expression [53].

#### 2.2.2. Sheep

Sheep production, like goat production, has seen significant growth in recent years, particularly in Mediterranean countries, Central Asia, and parts of Africa [54]. The sheep-milk industry has experienced growth, driven by its potential to produce high-value dairy products. For example, sheep dairies have been established in the United States for approximately 30 years, focusing primarily on the production of specialty cheeses [9]. The superior nutritional value of sheep milk compared to goat and cow milk, along with its high economic potential, has contributed to the growing interest in sheep dairy products [11]. The industry has also seen an increase in sheep-milk production, which is utilized in a variety of gourmet dairy products [55]. Despite this growth, there are indications of challenges and regional disparities. For instance, the Sardinian sheep dairy industry has faced a dramatic crisis, prompting investigations into ways to improve technical efficiency [56]. In contrast, New Zealand is exploring the potential to establish a competitive sheep dairy industry, suggesting that growth is not uniform across all regions [57].

According to the Food and Agriculture Organization, global milk production reached 10.3 million metric tons in the year 2017 [37]. Some of the main contributors were China, India, Australia, Nigeria, and Iran, where ovine production is an important part of the culture and traditions. The Mediterranean region is also characterized by ovine production, specifically with countries like Greece and Italy [58] producing premium-quality sheep cheeses, such as pecorino and feta [59]. In Europe, sheep-milk production is well-established and plays a fundamental role in the dairy market, while in the Middle East and North Africa, it contributes to food security [60]. Similar to goat production, sheep farming is crucial for rural economies and in regions where climatic, terrain, and drought conditions are unsuitable for bovine production [61]. In these areas, sheep meat, milk, dairy products, and textiles are the primary sources of income for many families, providing them with livelihoods and employment opportunities [62].

Sheep milk is rich in nutrients, particularly known for its high levels of β-carotene, vitamins A and E, conjugated linoleic acid, and fatty acids compared to other ruminants [63]. Like goat milk, the smaller fat globules present make it easier to digest for individuals with cow-milk sensitivities [64]. Sheep milk contains a variety of fats, including both saturated and unsaturated fatty acids. The composition of these fats can be influenced by factors such as the sheep’s diet, genetics, and environmental conditions. While saturated fatty acids are present in sheep milk, it also contains monounsaturated and polyunsaturated fatty acids, including omega-3 fatty acids and conjugated linoleic acid (CLA), which are considered beneficial for human health [65,66,67,68].

Volatile organic compounds (VOCs) are essential for the sensory experience of dairy products, as they influence flavor, aroma, quality, and even health benefits. Ruiz et al. (2023) explore the relationship between VOCs in sheep milk cheeses and their technological and sensory characteristics. This study highlights how different strains of lactic acid bacteria (LAB) affect aroma production, which in turn influences flavor profiles and the technological properties of cheese. Although the study does not directly address VOCs in sheep milk, it suggests that the selection of specific LAB strains could influence these compounds and thereby impact the sensory and technological characteristics of the final product [69].

In another study, S. Wang et al. (2023) identified 362 species of polar lipids in human and sheep milk, revealing significant differences in their composition. These differences may impact physiological functions and metabolic processes, suggesting that sheep milk has strong potential as a substitute for human milk due to its nutritional value [70]. Furthermore, Atti et al. (2006) found that sheep fed on pasture-based diets produce more milk with a better fatty acid composition compared to those fed in confinement. Pasture-based diets not only increase milk production but also improve its nutritional profile, making it more beneficial for human health [71]. These studies provide a comprehensive view of how different factors, such as bacterial strains, lipid composition, and diet, influence the quality and properties of sheep milk.

The relationship between VOCs in sheep milk and its technological and sensory characteristics is explored in Ruiz et al. (2023). The study focuses on the capacity of native and characterized LAB strains to produce aromas and identifies the VOCs responsible for flavor in cheeses made from raw sheep milk. Using a combination of Sanger sequencing for LAB identification and gas chromatography coupled with ion mobility spectrometry (GC-IMS), the research describes VOC profiles associated with different LAB strains and their influence on the cheese volatilome. The study reveals that LAB behavior and VOC production vary between cheese types, particularly in soft cheeses, and reports the presence of *Lactococcus lactis* subsp. *hordniae* in cheese for the first time [69].

Interestingly, while Ruiz et al. (2023) provide insights into VOCs in sheep-milk cheese and their connection to sensory properties, they do not directly address VOCs in sheep’s milk or their technological implications. However, the study suggests that selecting specific LAB strains can influence the VOC profile of sheep-milk cheese, affecting flavor and, potentially, its technological characteristics, such as texture and ripening behavior.

A study by S. Wang et al. (2023) identified a total of 362 species of polar lipids from 14 subclasses in mature breast milk (BM) and sheep milk (EM) through lipidomic analysis. Among these, 139 differentially expressed polar lipids (SDPLs) were selected, with 111 upregulated and 28 downregulated in EM compared to BM. Notably, the content of phosphatidylethanolamine (PE) (16:1_18:0) was significantly higher in EM than in BM, with a fold change (FC) of 69.5853 and a *p*-value < 0.0001. The study highlighted that sphingolipid metabolism and glycerophospholipid metabolism are critical pathways influenced by the observed differences in lipid composition. These findings suggest that variations in polar lipids between BM and EM could affect physiological functions and metabolic processes, providing insight for optimizing infant formula. The research also emphasizes the potential of sheep milk as a superior substitute for human breast milk due to its high nutritional value and polar lipid content [70].

On the other hand, Atti et al. (2006) describe an increase in milk production in sheep fed pasture-based diets (green barley and perennial ryegrass), as such sheep produced significantly more milk (617 mL/day) compared to those in confinement (363 mL/day). Although the confined sheep had higher fat (88.8 g/kg) and protein content (56.7 g/kg) in their milk, the total fat and protein yields were greater in the pasture groups. There was also a shift in fatty acid composition, with higher medium-chain fatty acids in the confinement group, while long-chain fatty acids increased in the pasture groups. The milk from the pasture groups had a higher proportion of C18:3 compared to the confinement group (4.5 vs. 2.7 g/1000 g). Additionally, the CLA content was higher in the milk from pasture-fed sheep (7.3 and 10.3 g/1000 g for GB and RG, respectively) compared to the confinement group (2.4 g/1000 g). This indicates that pasture-based diets improve the healthfulness of sheep milk and provide nutritional benefits, as such diets not only enhance milk production but also improve the nutritional profile of the milk, making it more beneficial to human health due to the higher concentrations of desirable fatty acids and CLA [71].

Label-free proteomic technology has identified differentially expressed proteins in sheep, goat, and cow milk. The proteins CTSB and BPIFB1 have been determined to potentially serve as markers for sheep milk [72]. The content of CTSB is higher in sheep milk than in cow or goat milk, as is BPIFB1, a secretion-related protein associated with immune response, with fungicidal and penetration-promoting capabilities, predominantly expressed in mucosal tissues [73] and highly expressed in sheep milk when compared to goat or cow milk. This could allow this technology to be used in identifying adulteration of dairy products by the inclusion of other species’ milk.

Additionally, proteomic changes in sheep milk due to subclinical mastitis have been investigated, identifying significant differences in the serum and membrane profiles of fat globules between infected and non-infected individuals, which could serve as biomarkers for the early detection of mastitis. The effect on the protein composition of milk was studied in samples from animals infected with *Staphylococcus chromogenes,* microbiologically negative animals with high somatic cell counts, and microbiologically negative animals with low somatic cell counts. The differentially expressed proteins were found to be related to signaling, biological regulation, response to stimuli, cell death, and immune processes such as inflammation and immune defense, among others [74].

Studies conducted on the crossbreed between the local Chinese Small-Tailed Han (STH) and DairyMeade breeds showed that differences in the proteomic profile of STH, F1, and F2 generations do not exhibit significant variations. Differential expression of the proteins WASHC4 and CUTA was detected between the F1 generation and STH, and of the proteins asLOC101105437, LOC101117231, SCGB2A2, and LOC101110099 between the F2 generation and STH. Serum proteins showed similar abundance across the three generations [75]. Proteomic studies have provided valuable insights into the protein composition of sheep milk and the identification of potential biomarkers for milk differentiation and disease detection, as well as information on physiological variations among different sheep breeds and generations. These findings contribute to the understanding of the nutritional composition of sheep milk and its processing into dairy products, with implications for breeding, dairy production, and quality control.

#### 2.2.3. Donkey

Unlike sheep and goat production, equine production of donkey milk is on a smaller scale, which has gained relevance in recent years in countries like China [76]. This production is fragmented and poorly regulated [77]. It is challenging to reliably quantify how many tons of equine dairy products have been produced in recent years [78]; however, it is known that production is concentrated in Europe, Asia, and Africa [79]. Donkey milk is generally lower in fat content compared to cow’s milk and human milk [80]. However, it is richer in certain nutrients. The lactose content in donkey milk is higher and more similar to human milk than cow’s milk [81]. Donkey milk also has a higher content of whey proteins, with a casein-to-whey-protein ratio of 52:37, which falls between human milk (lower) and cow’s milk (higher) [82]. Interestingly, donkey milk contains higher levels of some vitamins compared to cow’s milk. It has a higher vitamin C content, showing similarity to human milk in this respect [83]. Additionally, vitamins of the B-complex, including thiamine, riboflavin, niacin, pyridoxine, and folic acid, are found in higher concentrations in donkey milk compared to human milk [83]. In terms of amino acid profile, donkey milk contains higher percentages of essential amino acids (38.2%) compared to mare and cow milk [82]. It is particularly rich in serine, glutamic acid, arginine, and valine, while being lower in cystine [82]. The milk also contains higher levels of lysozyme, which contributes to its antimicrobial properties [82,84]. The lactose content can promote beneficial microbial proliferation in the intestinal tract [80]. The cholesterol content is 8.6 mg/100 g, accounting for 78.2% of the cholesterol present in human milk and 57.3% of that in cow’s milk [80]. Donkey milk is used in the production of cheeses, yogurt, and personal-care products [85] and is known for its hypoallergenic properties, attributed to its low total protein and casein content in the serum [86,87]. Products like donkey-milk soaps are sought after due to their good moisturizing capacity and anti-aging properties [85].

The casein fraction of donkey milk is more closely related to human homologs than to bovine ones, and the presence of multiple amino acid differences in the linear IgE binding epitopes when compared to those in cow’s milk makes donkey milk a valid natural substitute for cow’s milk in children with cow’s milk protein allergy (CMPA) [87].

Donkey milk is characterized by a low casein content, with values very close to those of human milk. According to a study conducted by Polidori and Vincenzetti, the total whey protein content in donkey milk varies between 0.49 and 0.80 g/100 g, while that of human milk ranges from 0.68 to 0.83 g/100 g. Among the whey proteins, the average concentration of α-lactalbumin in donkey milk is 1.8 mg/mL. The results of this study confirmed the potential for using donkey milk in the diet of children with cow’s milk protein allergy (CMPA) [88]. The presence of bioactive peptides such as lactoferrin, lactoperoxidase, and lysozyme, which exhibit antimicrobial activity and stimulate neonatal intestinal development, contributes to the nutritional profile of donkey milk for human neonate consumption [88].

A proteomic analysis has revealed that donkey milk contains a high concentration of amino acids, particularly serine, valine, arginine, and glutamate, along with a high concentration of essential amino acids in the milk’s protein fraction. It is particularly rich in β-lactoglobulin and lysozyme [82]. The essential amino acid content in donkey milk is higher than that found in mare and cow milk, which is important for human nutrition [82]. Furthermore, the ratio of casein to whey protein in donkey milk varies; one study reports a ratio of 52:37, which falls between the lowest value found in human milk and the highest in cow’s milk, while another study reports a ratio of 70.3:100 [80]. The proteomic analysis of donkey milk reveals a composition that is beneficial for human nutrition, especially for infants with CMPA. Its low allergenic potential, similarity to human-milk proteins, and rich content of bioactive peptides and essential amino acids make donkey milk a valuable alternative to cow’s milk. The variability in the casein-to-whey-protein ratio across different studies suggests that further research is needed to standardize these findings.

#### 2.2.4. Zebu

Domestic cattle descend from a now-extinct common ancestor, *Bos primigenius*, and are divided into two main types of breeds: humped (Bos indicus or *Bos taurus indicus*, including zebu) and non-humped (*Bos taurus*). The divergence between taurine and zebu cattle breeds is the result of distinct origins, selection, and adaptation events [89]. Zebu cattle are predominantly found in South Asia and Africa, but they have also been exported to South America, particularly Brazil. These animals are well-adapted to dry, hot climates, making them essential for production in the regions they inhabit [90]. Although their production volumes are lower than those of *Bos taurus*, zebu cattle are more resilient to harsh environmental conditions [91].

Genetic improvement of zebu is achieved by crossbreeding with cattle breeds like Holstein, aiming to combine the productive capacity of cattle with the environmental resilience of zebu [92,93]. Zebu cattle are primarily used for meat production, as well as for plowing and transportation, serving crucial roles for small-scale farmers. Crossbreeds between zebu and cattle, such as Holstein and Gyr, are selectively bred for cheese and powdered-milk production [94].

The proteomics of zebu milk, specifically *Bos indicus*, has been explored to understand genetic variation in milk protein loci and the differential abundance of milk proteins throughout the stages of lactation. Ceriotti et al. investigated genetic variability in four milk-protein loci in various *Bos indicus* and *Bos taurus* populations, as well as in their hybrids, revealing polymorphisms and a new synonymous variant in the *CSN2* gene. This study highlights the potential of milk-protein polymorphism to distinguish between zebu and taurine cattle, which could be useful for genetic improvement strategies and enhanced production [95]. Mol et al. present a comprehensive proteomic analysis of the bovine milk of the indigenous Indian Malnad Gidda cattle (*Bos indicus*) across different stages of lactation. A total of 564 proteins were identified, 403 of which showed differential abundance, providing insights into the biological processes and molecular functions relevant to each stage of lactation [96]. Milk composition and its dynamic nature in zebu milk, influenced by both genetic factors and physiological changes during lactation, should be taken into consideration for future applications.

#### 2.2.5. Yak

Yak husbandry provides milk, meat, fiber, and manure to its producers. In China’s Sichuan Province, yak milk is an important source of income and receives private funding for its development [97]. Yak rearing is common in regions of India [98], localities of the Tibetan–Qinghai Plateau [99], and Nepal [100]. Although yak-milk production is lower than that of cattle, it is attractive due to its composition and nutrients, including a higher protein concentration compared to human and cow milk [101]. Some characteristics that make yak an appealing animal for productive environments are its resistance to hypoxia and fatigue [102].

A proteomic analysis of yak milk revealed a composition rich in proteins with nutritional and therapeutic value. Studies have identified a wide range of proteins, including those related to the immune system and involved in redox processes and ATP binding; some of these proteins are differentially expressed between yak breeds [101]. The proteomic patterns of yak milk show significant differences compared to other species, like cows, buffalo, goats, and camels, with certain proteins serving as species-specific markers. In the case of yak milk, an uncharacterized soluble protein (AC: F1MK50) is highly abundant [103]. Additionally, the thermal stability of yak milk proteins has been characterized, indicating a pH-dependent behavior and interactions between caseins and whey proteins when heated above 65 °C [104,105]

It is interesting to note that, although yak milk is not produced in large quantities, it is superior in nutritional composition compared to cow milk, particularly in regard to protein and fat content [102,106]. The metabolic mechanisms during the yak lactation cycle have been studied, revealing variations in metabolites and impacted pathways such as the Krebs cycle and amino acid metabolism [106]. Additionally, genetic expression patterns related to milk synthesis in yaks have been evaluated, highlighting the importance of amino acid and glucose transporters, and insulin signaling through the mTOR pathway [107]. These findings contribute to understanding the nutritional benefits of yak milk, its thermal stability characteristics, and the underlying metabolic and genetic mechanisms during lactation, as well as valuable insights for improving milk yield and quality for yak-milk products.

#### 2.2.6. Buffalo

The water buffalo, colloquially known as the water bison (although they are not the same species), is divided into two subspecies: river and swamp [108]. It was domesticated in India and China between 2500 and 1400 BC [109]. It is commonly raised in Asian and African countries, such as Turkey [110], Nepal [111], China [112], India [113], and Egypt [114]. Additionally, it was introduced to Brazil in 1895 [109] and is used as a source of food and as a work animal. Its milk is used to produce soft cheeses, mainly mozzarella [115,116].

Swamp buffaloes are raised as draft animals but are also capable of producing up to 600 kg of milk per year [117]. They are known for their ability to convert low-quality feed; adaptability to adverse environmental conditions, with breeds capable of enduring tropical conditions (high heat and humidity, such as in floodplains); and ability to survive in various terrains and vegetation [118]. In tropical and subtropical countries, they are a key part of the agronomic trade. Recent studies show that breeds raised in high-heat conditions have developed resistance to high temperatures, unlike those in their natural habitat, making them a promising animal for production amid climate change [119].

A proteomic analysis of buffalo milk has been carried out to understand its protein composition and functional properties. Techniques like proteomic and lipidomic analyses, isobaric tags for relative and absolute quantification (iTRAQ), and two-dimensional gel electrophoresis with mass spectrometry have been used to elucidate the buffalo milk proteome [120,121,122]. The most abundant proteins in buffalo milk are caseins and whey proteins. Specifically, the main caseins identified are αS1-, αS2-, βγ-, and κ-casein, with respective proportions of total casein content at 32.2%, 15.8%, 36.5%, and 15.5% [123]. The calculated casein-to-protein ratio is 83.74 ± 2.1%, consistent with previous research. Additionally, the β-lactoglobulin content is approximately 1.3 times higher than that of α-lactalbumin [123]. Buffalo milk also contains a favorable ratio of essential and non-essential amino acids, contributing to the high nutritional quality of buffalo dairy products [124]. The presence of other bioactive components and the lipid profile of the milk further enhance its overall quality and potential health benefits.

Interestingly, despite general similarities in the protein composition of milk from different species, buffalo milk has been shown to exhibit distinct proteomic patterns. For example, clusterin has been identified as a characteristic protein in buffalo milk. Additionally, the presence of specific proteins, such as β-lactoglobulin and α-lactalbumin, has been used to detect adulteration of buffalo milk with other types of milk, highlighting the specificity of the buffalo-milk proteome [120,121].

#### 2.2.7. Camelids

In recent years, the camel-milk industry has advanced significantly, notably with the implementation of mechanical milking and the creation of large-scale dairy farms [125]. This progress has increased the demand for camel milk and its derivatives, driving more research and development in areas like milk quality, product development such as yogurt [126,127], probiotic beverages [128], and processing technologies [129]. There is even potential for its use in infant formula production [130] and highly nutritious foods [131]. Notable characteristics of camel milk include antioxidant potential, glycemic regulation, and anti-inflammatory activity [131,132]. However, one of the main challenges is its salty taste [133].

The proteomic analysis of camel milk reveals a unique composition that distinguishes it from the milk of other mammals. Girardet et al. detail the characterization of proteose peptone 3 (PP3) from camel milk, a protein that is part of the family of glycosylation-dependent cell-adhesion molecules 1 (GlyCAM-1), which may play an immunological role in newborn camels [134]. This protein contains an insertion in the N-terminal region and potential O-glycosylation and phosphorylation sites. Yang et al. use iTRAQ techniques to analyze the serum proteome of various species, including camel milk, identifying 211 proteins and highlighting significant proteomic differences between camel milk and other species [103]. El-Hatmi et al. focus on the variation in the composition of whey proteins in camel milk and colostrum over time, noting the presence of immunoglobulins and the absence of β-lactoglobulin, a protein commonly found in cow’s milk, which is often associated with cow’s milk protein allergy (CMPA). This makes camel milk a potential alternative for producing “humanized” milk [135]. Saadaoui et al. identify key proteins in the milk fat globule membrane (MFGM) in camel milk, providing insight into the formation of lipid droplets in mammary epithelial cells [136].

Although these studies offer a comprehensive view of the proteomics of camel milk, they also reveal contradictions regarding the presence of certain proteins. For instance, El-Hatmi et al. report the absence of β-lactoglobulin in camel milk, a protein commonly found in cow’s milk, but this is not explicitly mentioned in other studies [135]. Moreover, the proteomic patterns and specific proteins identified as characteristic of camel milk in Yang et al., such as acidic whey protein and quinone oxidoreductase, are not discussed in the context of their functional roles nor compared with proteins identified in other works [103]. Despite these inconsistencies, the presence of unique proteins and the absence of others, like β-lactoglobulin, suggest a specialized adaptation of camel milk to its environment and potential health benefits.

#### 2.2.8. Reindeer

The production of deer milk is receiving attention for its potential in cheesemaking and as a source of high-quality nutrition [137,138]. Deer milk, particularly that from red deer, is notable for its high-fat, -protein, and -mineral content, as well as its unique gelling properties [138], and has been proposed as a highly nutritional food for individuals with low body mass index (BMI) [139].

Doe milk contains higher levels of proteins, fats, and certain minerals compared to cow’s milk, with unique compositional changes throughout lactation [138]. In vitro digestion studies have demonstrated that doe-milk proteins are more digestible and generate more peptides than cow-milk proteins [140]. Ha et al. observed that the caseins in red deer milk exhibit greater mobility in 1D-SDS PAGE compared to those in cow, sheep, and goat milk, although their retention profile in RP-HPLC is similar. The proportions of α-La and β-Lg are also distinct in red deer milk, with different behaviors observed in their ammonium sulfate fractionation and anion exchange chromatography compared to other species [141].

#### 2.2.9. Llamas and Alpacas

The llama (*Lama glama*) and alpaca (*Vicugna pacos*) are herbivores native to South America, domesticated from their wild ancestors, the guanaco (*Lama guanicoe*) and vicuña (*Vicugna vicugna*), respectively, between 6000 and 7000 years ago [142]. The llama dairy industry has a rich history, as the llama has been used for various purposes by Andean peoples for centuries [143]. In the case of the alpaca dairy industry, it is important for low-income families, highlighting the need for improved state capacity and support for small producers. Alpaca milk can be used to produce cheese with favorable physicochemical properties and sensory characteristics [144]. The potential of camelids, including alpacas, is noteworthy, as they may become an important source of protein in the face of climate change [145].

Proteomic studies have provided insight into the composition of llama, alpaca, and other mammal milks. Proteins identified in llama milk, such as caseins, α-lactalbumin, lactoferrin, and serum albumin, have been detected using various proteomic techniques [142]. A study found that the average values for the variables in alpaca and llama milk were similar, except for pH, during the 40-to-45-day lactation period, indicating that the main components do not vary throughout lactation [142]. The total solids content in alpacas is 16.03%, and in llamas, it is 16.41%, slightly higher than human milk (12–13% on average), while the fat content was 3.42% in alpacas and 3.65% in llamas [142]; however, tearlier research reported values of 4.55% for alpacas [146]. The protein content was 4.59% in alpacas and 4.65% in llamas [142], higher than the protein content in other ruminants and human milk. The lactose content was 6.9% in alpacas and 6.99% in llamas, higher than the lactose content in other domestic ruminants [142].

## 3. Characteristics of Milk Produced by Non-Traditional Animals

Milk is a complex biological fluid that contains essential nutrients for growth and development. The main molecules in milk include proteins, lipids, and carbohydrates, with each playing a crucial role. Proteins, particularly caseins and whey proteins, contribute to the formation of micelles and have various functions, such as enzymatic activity and antimicrobial properties [147]. Lactoferrin, a whey protein, exhibits numerous biological functions, including antibacterial, antiviral, and immunomodulatory effects [148]. Lipids in milk are emulsified in the form of large globules, while lactose is the primary sugar. Milk also contains oligosaccharides, which play protective roles. Advanced analytical techniques such as proteomics, glycomics, and lipidomics have expanded our understanding of the bioactive components of milk, allowing for a comprehensive analysis of these molecules [149]. This knowledge is crucial for nutritional research and the development of beneficial food products. Below is a list of some key milk molecules and their main characteristics:

- Caseins: Caseins are phosphorylated and glycosylated proteins that are composed of four main fractions (αS1-, αS2-, β-, and κ-casein). They are primarily associated with the formation of casein micelles, which affect the firmness and yield of dairy products, mainly cheeses. About 95% of caseins form micelles, with the remainder serving as soluble caseins. Caseins are one of the main allergenic proteins in milk [150].
- α-Lactalbumin: α-Lactalbumin is a globular protein that constitutes approximately 20% of human milk proteins and 3.5% of bovine milk proteins. It serves as a cofactor in lactose synthesis and plays a role in calcium ion transport [151].
- β-Lactoglobulin: β-Lactoglobulin is s globular whey protein present in both human and animal milk. It releases bioactive peptides that perform hormone-like functions [152]. It is associated with cheese coagulation capacity [153].
- Cholesterol: Cholesterol is an essential component of milk, necessary for cell membrane formation and the synthesis of steroid hormones [154]. Human milk is rich in cholesterol, which has been proposed to be an important component in infant formula [155].
- Lactose: Lactose is the main carbohydrate in milk, playing crucial roles in maintaining osmotic balance in the mammary gland and contributing to the energy value of milk [156]. It also supports the balance of intestinal microflora and enhances calcium absorption [157].

**Figure 2:**
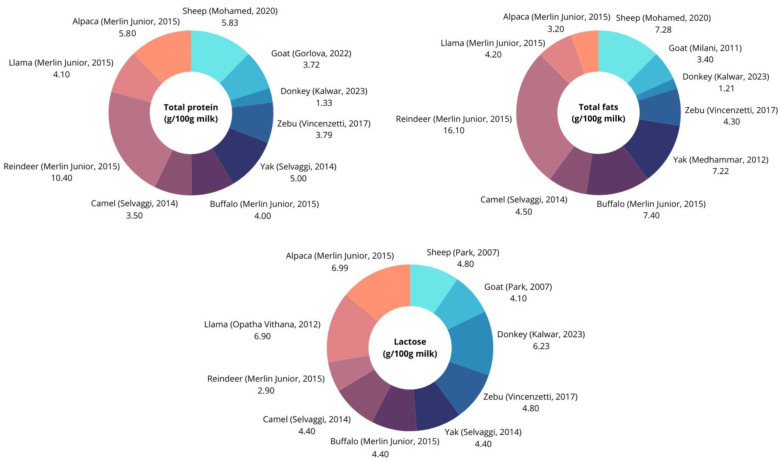
Graphs displaying the total protein, fat, and lactose content in the milk of sheep, goats, donkeys, zebus, yaks, buffalo, camels, reindeer, llamas, and alpacas. Total protein from sheep [158], goat [157], donkey [159], zebu [160], yak [161], buffalo [162], camel [161], reindeer [162], llama [162] and alpaca [162]; total fats from sheep [158], goat [27], donkey [159], zebu [160], yak [163], buffalo [162], camel [161], reindeer [162], llama [162] and alpaca [162]; and total lactose from sheep [164], goat [164], donkey [159], zebu [160], yak [161], buffalo [162], camel [161], reindeer [162], llama [162] and alpaca [162] are shown. Data is presented as grams per 100 g of milk, with values sourced from the indicated references.

Some of the main components of non-traditional animals’ milk are presented in Figure 2. More detailed information on both major and minor components are shown in Table 1.

## 4. Factors Influencing Milk Production and Quality in Minor Livestock

Emerging technologies in milk processing aim to retain immunoactive components and ensure microbial safety while minimizing the negative effects of thermal processing [175,176]. Non-thermal methods, such as ultraviolet light, ultrasonication, and high-pressure processing, are gaining interest for their ability to preserve thermosensitive components, like lactoferrin and immunoglobulins [175]. Other promising technologies include pulsed electric fields and plasma technology [177]. These methods can achieve microbial reduction with minimal thermal input, preserving the nutritional and sensory qualities of milk [178,179]. Additionally, new thermal technologies, such as ohmic heating, are being explored [178]. These emerging technologies offer opportunities for diversifying milk fat products and enhancing nutritional and functional attributes [180]. However, consumer perception and acceptance of these novel processing methods remain important considerations for future innovations in the dairy industry [178]. A summary of some of the currently investigated technologies is presented in Table 2.

- High-pressure processing (HPP): HPP is a non-thermal food-preservation technique that applies high pressure (100–1000 MPa) to food products at or near room temperature [209,210]. This innovative technology inactivates pathogenic and spoilage microorganisms while maintaining the sensory and nutritional attributes of fresh, preservative-free, minimally processed foods [209,211]. HPP has been used in many animals’ milk, including cow’s, goat’s, sheep’s, donkey’s, yak’s, buffalo’s, and camel’s milk. For goat’s milk, HPP has been used “as a gentle preserving method” that has a positive impact on the quality and safety of the product, making it possible to store treated samples for up to 10 days at 4 °C [179]. Interestingly, HPP treatment of goat’s milk resulted in significant changes in lactose content and a reduction in fat globule size, which could be advantageous for cheesemaking and other dairy-product industries [212]. In the case of sheep’s milk, which has been shown to have baroprotective characteristics, an HPP treatment successfully inhibited *E. coli* and *Pseudomonas fluorescens* to less than 6-log cfu/mL at 50° [181] and has been shown to retain the anti-rotaviral activity of sheep derived buttermilk and whey [182]. The use of HPP in donkey milk ensured the death of E. coli, as it was not detected in culture from day 0 onwards [183]. In an experiment conducted by Feng et al., powdered yak milk was treated by different methods, including HPP [184]. Finally, in buffalo milk, HPP was implemented and altered the casein micelle size slightly at levels of 100–600 Mpa for 30 min, and it altered this fraction significantly (35%) at pressures of 800 MPa. This study suggests that HPP treatment affects many constituents, such as calcium, lactalbumin, and caseins, which could be observed in other species’ milk, although at different pressures [185]. The primary reasons for using HPP in milk processing include microbial inactivation, shelf-life extension, and modification of milk components for improved functionality. Overall, HPP can effectively reduce bacterial concentrations, including pathogenic *E. coli*, *Salmonella*, and *L. monocytogenes* [213]. It also affects milk proteins, causing disruption of casein micelles, denaturation of whey proteins, and solubilization of minerals associated with the micelles [210]. These changes can lead to improved rennet coagulation time, increased cheese yield, and altered functional properties in dairy products [211].
- Pulse electric field (PEF): Pulsed electric field (PEF) technology has several important applications in the dairy industry. PEF is primarily used as a non-thermal preservation method for milk and dairy products, offering an alternative to traditional heat treatments, like pasteurization and sterilization. It can effectively inactivate pathogenic and spoilage microorganisms while minimizing changes to the nutritional, functional, and organoleptic qualities of dairy products [214,215]. The technology has shown promise in extending the shelf life of dairy products while maintaining fresh-like characteristics [216,217]. Furthermore, PEF has applications beyond microbial inactivation. It has been found to improve probiotic retention in spray-dried powders and assist in dairy-waste treatment [218]. Additionally, PEF technology has shown potential in enhancing various dairy-processing operations, including homogenization, emulsification, crystallization induction, fat separation, and improved functional properties of whey proteins and dairy powders [219]. In the case of goat milk’s pasteurization, a short exposure of milk to low-intensity PEF and thermal treatment at 63 °C caused the same reduction in the population of *L. monocytogenes* when compared to 72 °C treatment without PEF; however, it was found to induce heat resistance in the surviving population [187]. As for *E. coli*, pretreatment with PEF reduced viable cells by 7.5-log (CFU∙mL^−1^) compared to 4.6 ± 0.198-log (CFU∙mL^−1^) without PEF treatment [187]. In summary, while PEF technology shows considerable potential for use in liquid dairy products, particularly for microbial inactivation and shelf-life extension, its commercial adoption in the dairy industry remains limited. Further research is needed to optimize PEF processing parameters, enhance treatment system designs, and evaluate its effects on product quality and safety at an industrial scale [220,221]. The technology’s ability to preserve the natural characteristics of dairy products while ensuring safety makes it a promising area for future development in the dairy industry.
- Ultrasound processing (USP): USP has numerous applications in the dairy industry, offering potential benefits over conventional methods. It can be used for inactivation of microbes and enzymes, homogenization, emulsification, creaming, crystallization, and functionality modifications within dairy systems [222]. The technology is particularly effective in eliminating pathogenic microorganisms, such as *Salmonella*, *E. coli*, *S. aureus*, and *Listeria monocytogenes*, improving food safety and shelf life [223]. Interestingly, it can also enhance the fermentation process of lactic acid bacteria by modifying their metabolic activity, reducing fermentation time, and improving the quality characteristics of fermented milk products [224]. This dual nature makes ultrasound a versatile tool in dairy processing. This method has been tried in sheep’s, goat’s, donkey’s, buffalo’s, and camel’s milk. In sheep’s milk, the effect of USP on microbiological quality and protein and amino acid profiles was tested, and no relevant change was reported on amino acid profile, while it maintained acceptable amounts of lactic bacteria and eliminated other microorganisms [188]. USP improved microbiological quality in goat cheeses and inactivated coagulase-positive staphylococci, as well as the unsaturated acid proportion and the AI, TI, and H/H lipid quality; and it also reduced the cholesterol content [189]. Caseins from donkey’s milk were identified as sensitive to ultrasonic treatment’s stronger than 200 W, although the whey fraction was not affected from 200 to 600 W. After treatment, fermentation products and the texture of the cheese were soft, associated with the lower concentration of caseins [190]. In buffalo milk, USP with high power input had an important effect on milk homogenization and reducing the growth rate of coliform bacteria [191]. Finally, the use of USP in camel milk at 160 W for 10 min completely reduced the microbial count and retained bioactive properties such as antioxidant properties and particle distribution [192]. In conclusion, ultrasound technology has emerged as a promising alternative in dairy processing, offering advantages such as improved product properties, cost savings, and energy efficiency [222]. It has been applied in the processing of various dairy products, including raw milk, cream, yogurt, butter, ice cream, and cheese [225]. However, it is important to note that while ultrasound offers many benefits, factors such as sonication time, temperature, and frequency need to be carefully controlled to optimize results and minimize potential drawbacks, like the production of off-flavors [226].
- UV-C (UV): UV-C radiation is a form of ultraviolet light with wavelengths between 200 and 280 nm, known for its germicidal properties [227,228]. It is an effective method for inactivating pathogenic and spoilage microorganisms in milk and dairy products by forming lesions in DNA and/or damaging cellular enzyme activity and cytoplasmatic membrane integrity [227], despite its low transmittance within opaque liquids [193]. The dairy industry can utilize UV-C technology as a non-thermal alternative to traditional pasteurization methods. It offers several advantages, including low maintenance and installation costs, minimal energy use, and food preservation without some undesirable effects of heat treatments [227,229]. UV-C can be applied to disinfect air, water, food-contact surfaces, and packaging materials in dairy processing [230]. It can also be integrated with existing pasteurization techniques to reduce pasteurization temperature, potentially improving the sensory properties of milk [231]. A study by Ansari et al. evaluated the effectiveness of UV pretreatment in enhancing thermal inactivation of *Bacillus subtilis* spores in skim cow milk, whole cow milk, and whole sheep milk. Researchers applied UV pretreatment (D Act 2.37 ± 0.126 J/mL), followed by thermal treatment at 110 °C for 30 s. This approach achieved spore reductions of approximately 6-log CFU/mL in skim cow milk, 2.9-log CFU/mL in whole cow milk, and 1.1-log CFU/mL in sheep milk. The results suggest that UV combined with heat may offer an alternative to traditional UHT treatment (135 °C, 3 s) for sterilizing skim milk at lower temperatures, preserving some of its desirable characteristics [193]. Kasahara et al. examined the efficacy of pulsed UV light for inactivating *E. coli* in goat milk and assessed its impact on organoleptic properties. Goat-milk samples inoculated with *E. coli* were treated with various doses of pulsed UV light from an exciplex laser unit. A 6-log reduction in microbial load was achieved with a dose of 10,000 mJ cm⁻^2^. Aromatic changes were observed at 5000 and 10,000 mJ cm⁻^2^ compared to non-irradiated samples, though no significant differences were found in physical or compositional attributes between irradiated and control samples. In regard to donkey milk, a study assessed UV-C’s effectiveness in inactivating six foodborne pathogens in raw donkey milk. The milk was inoculated with *L. innocua*, *S. aureus*, *B. cereus*, *Cronobacter sakazakii*, *E. coli*, and *Salmonella enteritidis* and then exposed to UV-C doses up to 1300 J/L. *L. innocua* showed the highest UV-C resistance, requiring 1100 J/L for complete inactivation, while other pathogens were effectively inactivated within the range of 200–600 J/L [195]. Dasalkar et al. examined the impact of UV-C on microbial inactivation, amino acid profiles, metabolite functional groups, and physicochemical properties of cow and buffalo milk. Cow- and buffalo-milk samples inoculated with *E. coli*, *S. enterica*, and *L. monocytogenes* were treated with UV-C doses of up to 4.6 J cm⁻^2^, achieving log reductions of 4.07, 4.41, and 4.92 in cow milk and 3.71, 3.74, and 4.42 in buffalo milk, respectively. While UV-C treatment caused no significant changes in amino acid profiles, metabolite functional groups, or sensory properties, a notable color change was observed. These findings support UV-C as a potential non-thermal treatment for microbial reduction in milk [196]. Finally, experiments in camel milk assessed the impact of varying UV-C doses on *E. coli* O157 and *Salmonella enterica* serovar Typhimurium viability, as well as on the chemical properties of camel milk. Pasteurized camel-milk samples, inoculated with the pathogens, were treated with UV-C in a continuous flow system. UV-C doses of 4.15, 8.30, and 12.45 mJ/cm^2^ achieved *E. coli* reductions of 1.9-, 3.3-, and 3.9-log; and *S. Typhimurium* achieved reductions of 0.9-, 3-, and 3.9-log, respectively. Secondary lipid peroxidation levels, protein profiles, and major milk components remained stable after treatment, although changes in conjugated linoleic acid isomers and three new volatiles were detected. The 12.45 mJ/cm^2^ dose did not achieve the FDA’s 5-log pathogen reduction standard, but UV-C treatment minimally affected camel-milk components. These findings suggest UV-C light as a viable non-thermal approach for reducing spoilage bacteria and pathogens in raw donkey milk. Interestingly, recent developments in UV-LED technology offer the potential for customized and point-of-use disinfection products in the dairy industry [228]. However, it is important to note that while UV-C is effective in reducing microbial counts, high-dose treatments may be detrimental to the sensory properties of milk [231]. Therefore, optimizing process parameters such as exposure time, UV dose, and equipment design is crucial for maintaining product quality, while also ensuring effective microbial inactivation [227,232].
- Gamma radiation (GR): Gamma radiation is a penetrating form of electromagnetic radiation arising from the radioactive decay of atomic nuclei, consisting of the shortest-wavelength electromagnetic waves and imparting the highest photon energy [233]. It is one of the three types of ionizing radiation used for food processing, along with accelerated electrons and X-rays [234]. In the dairy industry, gamma radiation can be used as a non-thermal processing technology to destroy micro- and macroorganisms in food products. This technique aims to maintain quality, improve safety, and reduce post-harvest loss while retaining desired organoleptic characteristics and health benefits [235]. Gamma radiation has the potential to reduce allergenicity and provide sterile diets for immunocompromised patients [235]. Interestingly, despite its potential benefits, the use of irradiation as a preservative technique for dairy products has received little attention due to the complexity of product varieties [235]. While accepted in some countries, the adoption of irradiation faces strict opposition in others due to environmental and health safety concerns [234,235]. Consumer acceptance of irradiated foods depends on proper food labeling and processing information [234]. Sheep butter made from gamma-irradiated cream (1 kGy) was analyzed for 90 days of refrigerated storage. The irradiated butter had a 10% higher water content than control butter and showed a slower bacterial growth rate, though fungal growth was similar across both types. Sensory assessments indicated general acceptance of irradiated butter by panelists. While gamma irradiation reduced the microbial load in the cream, it did not extend the shelf life of the resulting butter, as traditional manufacturing processes contributed significantly to microbial contamination [198]. For goat’s milk, in a study by Fohely et al., 30 samples were irradiated with gamma rays, resulting in a reduction of viability in pathogens, but altering the protein structure of basic aromatics acids (tyrosine and tryptophan) compared to X-rays [199]. In camel’s milk, escalating doses of irradiation led to a 3–5-log reduction in microbial count. A 9 kGy dose caused a minor 7.4% decrease in antibody activity and a 13% reduction in total camel IgG, with no impact on major whey proteins (lactoferrin, casein, and α-lactalbumin), as confirmed by SDS-PAGE. Thus, a 9 kGy dose effectively sterilizes camel whey powder by reducing bacterial load with minimal effects on antibody function and protein integrity [200]. Overall, gamma radiation offers a promising non-thermal alternative to traditional thermal treatments in the dairy industry. However, its application remains limited due to safety concerns and consumer awareness. Further research and education are needed to explore its full potential and address these challenges in the dairy-processing sector [234,235].
- Microfiltration (MF): Microfiltration (MF) is a pressure-driven membrane-separation process widely used in the dairy industry for various applications. It employs membranes with pore sizes typically around 0.1 μm to separate particles and bacteria from milk and other dairy products [236,237]. In the dairy industry, MF has been applied since the late 1980s, with developments like ceramic membrane technology and the Uniform Transmembrane Pressure (UTP) system [238]. It is used for several purposes, including the removal of bacteria and spores from skim milk to extend shelf life [237], separation of casein micelles from soluble proteins [239], and fractionation of milk proteins and fat [240]. It is important to consider that the process can be affected by factors such as ionic strength, which influences the behavior of casein micelles and the formation of deposits on the membrane surface [236]. In the case of sheep’s milk, a study assessed the effects of microfiltration (1.4 μm ceramic membranes, 50 °C) on partially defatted ovine (0.4% fat) and bovine (0.3% fat) milk. The results showed a reduction in the total mesophilic bacteria by 4-log in ovine permeate and 2-log in bovine permeate, improving microbial quality. Protein and total solids contents were significantly reduced in permeates (*p* < 0.05). RP-HPLC analysis indicated decreased αs1- and β-casein but increased κ-casein in permeates. Alkaline phosphatase activity followed fat allocation, while cathepsin D activity remained stable despite somatic cell removal. Curd firmness in ovine milk showed no significant difference between feed milk and permeate, suggesting that microfiltration could serve as a pretreatment step for ovine milk in cheesemaking [201]. As for goat’s milk, a study evaluated the microbiological, physicochemical, and functional quality of a goat-whey orange-juice beverage (GOB) processed by microfiltration (0.2 µm) at varying temperatures (20–50 °C) and compared to conventional LTLT heat treatment (63 °C for 30 min). Microfiltration at temperatures of 30 °C or higher effectively reduced microbial counts (AMB, molds, yeasts, and LAB). Microfiltration temperature minimally impacted pH, color, volatile compounds, and bioactive compounds but influenced rheological parameters, with 20–30 °C maintaining consistency similar to LTLT treatment. Mild microfiltration temperatures (30–40 °C) are recommended to achieve microbial quality and preserve texture, functional properties, and volatile compounds [202]. Considering this, MF is a relevant technology in the dairy industry, offering benefits such as improved product quality, extended shelf life, and the development of new dairy products. Its applications continue to expand, making it an essential tool for dairy processors in meeting consumer demands and improving overall efficiency [241,242].
- Cold plasma processing (CPP): Cold plasma processing is a non-thermal food-processing technique that has gained popularity due to its environmentally friendly and more economical nature [243]. It involves the generation of reactive species without significantly raising the temperature of the treated product, making it an effective method for maintaining food safety while preserving nutritional and sensory attributes [243,244]. In the context of milk products, cold plasma is used for several reasons: microbial decontamination, as CPP can effectively inactivate microorganisms in milk and dairy products, ensuring food safety without the need for high-temperature treatments [244,245]; protein modification, as CPP can enhance the functional properties of milk proteins, such as water-holding capacity, solubility, and foaming capacity [246]; and quality preservation, because, unlike thermal processes, cold plasma can maintain milk safety while minimizing negative impacts on quality attributes such as protein denaturation, non-enzymatic browning, and vitamin loss [245]. However, it is important to note that cold plasma treatment can have both positive and negative effects on milk products. While it can improve certain functional properties and reduce microbial load, it may also lead to lipid oxidation, protein aggregation, and off-flavors if not optimized properly [243,245]. Additionally, cold plasma treatment can alter the microbiota and volatile flavor compounds in milk, potentially affecting its sensory characteristics [247]. In a study by Wang et al., they evaluated cold plasma-processing times of 30 s, 180 s, and 300 s on microbial inactivation, physicochemical properties, and protein structure of raw sheep milk, with pasteurized milk as a control. The microbial inactivation of 300 s matched that of pasteurization, with smaller casein micelle size and polydispersity (*p* < 0.05). Increasing the processing time reduced the pH (6.79 to 6.65), color a* value (−1.74 to −1.98), and freeze-dried moisture (from 43.4 g/kg to 28.9 g/kg). Scanning electron microscopy revealed a more uniform protein dispersion in 300 s, supporting cold plasma as a promising pasteurization alternative [203]. Another study on goat milk evaluated the effects of High-Power Ultrasound (HPU) and Gas-Phase Plasma (GPP) treatments on fatty acid composition and sensory properties of goat milk. Both treatments reduced monounsaturated fatty acids (MUFAs) from 16.9% to 14.5% and polyunsaturated fatty acids (PUFAs) from 8.3% in the control to 2.7–5.4%, depending on parameters. These changes were absent in pasteurized samples. Sensory evaluation indicated significant impacts (*p* < 0.05), with HPU (over 6 min) and GPP imparting “foreign-metal,” “burnt,” and “ozone” odors, rendering the milk unpalatable. Overall, HPU and GPP decreased MUFA and PUFA while increasing saturated fats, resulting in undesirable sensory qualities [204]. In conclusion, cold plasma processing offers a promising alternative to conventional thermal treatments for milk products. Its ability to ensure food safety while maintaining quality makes it an attractive option for the dairy industry. However, careful optimization of process parameters is crucial to maximizing the benefits while minimizing potential negative impacts on product quality [245,248].
- Ohmic heating (HP): Ohmic heating, also known as Joule heating or electrical resistance heating, is a thermal processing method that generates heat by passing an electric current through a food material [249,250]. It is used in the dairy industry as an alternative to conventional heating methods due to its ability to provide rapid and uniform heating of liquid, semi-solid, and particulate foods [251,252]. In dairy processing, ohmic heating is particularly useful for treating viscous products like yogurts, providing a basis for novel dairy-product structuring and significantly reducing fouling and corrosion of heating equipment [252]. It offers several benefits, including faster and more uniform heating, improved nutritional quality, effective inactivation of enzymes and microbes, and reduced processing time compared to conventional methods [253]. Additionally, ohmic heating can maintain the color and nutritional value of food while allowing for continuous processing of particulate foods [249,254]. In a study in sheep milk, the energy consumption and microbiota impact of ohmic heating (OH) versus conventional heating (CH) for pasteurizing fresh and thawed sheep milk over 15 days at 4 °C were tested. OH pasteurization using 8.33 and 5.83 V/cm reduced energy use by 72–73% compared to CH (515 KJ). Both methods achieved at least 4.2-log reductions in bacterial load. Amplicon sequencing showed *Staphylococcus* dominated in raw milk initially, while *Pseudomonas* became most prevalent after 15 days of refrigeration. Bacterial composition remained consistent between OH and CH treatments during storage [205]. A study by Pereira et al. examined the effects of ohmic heating versus conventional heating on the inactivation kinetics of *Escherichia coli* in goat milk and *Bacillus licheniformis* spores in cloudberry jam. Ohmic heating reduced the decimal reduction time (D) for *E. coli* in goat milk compared to conventional methods and lowered the z-value, indicating a smaller temperature increase needed for a ten-fold reduction in D [206]. Another study compared the microbial inactivation effects of ohmic and conventional heating on buffalo milk under identical temperatures, assessing total plate count, yeasts, molds, coliforms, *E. coli*, and *Salmonella*. Milk samples treated with ohmic heating showed significantly lower microbial counts than those treated conventionally, with *Salmonella* completely inactivated by ohmic heating. Paneer made from ohmically heated milk also exhibited reduced hardness compared to that from conventionally heated milk. These results suggest that ohmic heating is an effective technique for buffalo-milk pasteurization [207]. As for camel milk, a study compared ohmic and conventional pasteurization effects on the microbial and nutritional quality of camel milk at 63 °C for 3.75, 7.5, 15, and 30 min. Ohmic heating reduced the total bacterial count from 2.35- to 1.83-log CFU/mL, with a resulting pH of 6.54, 88.19% moisture, 3.09% fat, 2.43% protein, 3.25% lactose, and 0.85% ash. Conventional heating reduced bacterial counts similarly, from 2.37- to 1.87-log CFU/mL, yielding a pH of 6.6, 87.66% moisture, 2.99% fat, 2.33% protein, 3.16% lactose, and 0.84% ash. Both methods were effective, with ohmic heating offering slight variations in nutritional retention [208]. However, ohmic heating also presents some complications. Most studies associate it with high energy costs, and its use is limited to food products with appropriate electrical conductivity [255]. The process can increase free fatty acids, apparent viscosity, and hydroxymethylfurfural content in concentrated milk, while decreasing pH value and whiteness [251]. Furthermore, the higher initial cost, lack of applications in foods containing fats and oils, and limited awareness are factors that restrict its widespread use in the food industry [256]. Despite these challenges, ohmic heating remains a promising technology for improving the sustainability and efficiency of dairy-processing operations [252].

## 5. Perspectives and Challenges

The quality of milk from non-traditional animals will be influenced by the processing technologies implemented, potentially altering its nutritional composition and functional properties either positively or negatively [257]. For example, goat milk, with its low concentration of αs1-casein and high concentration of β-casein, presents challenges during the manufacturing process [258]. The processing technologies used for milk from these animals are generally the same as those applied to the milk and dairy products of other animals. However, goat and sheep milk do not require homogenization due to their natural characteristics [259]. Conventional thermal processing technologies are highly effective in reducing the presence of bacteria, fungi, and yeasts in goat milk [260], increasing its shelf life. However, these processes negatively affect sensory characteristics, such as color and flavor, primarily due to changes in the saturated fatty acid fraction [261]. Additionally, thermal technologies are energy-intensive [187,261], prompting interest in finding processing methods that reduce microbial loads without altering product composition. Common thermal technologies include ohmic resistance heating [262], microwave heating [263], and pasteurization. Non-thermal technologies include pulsed electric fields [264], hydrostatic pressure [265], micro- and ultrafiltration [266], ultrasonication [263], UV-light treatment [227], and CO₂ packaging/processing [267]. It is essential to determine the specific requirements for the milk of each species to enhance its characteristics and increase its market appeal as an alternative to traditional products.

Technologies like these face challenges in implementation within the small-animal dairy industry for several reasons: These advanced processing technologies often require significant initial investments in specialized equipment and infrastructure, which may be prohibitively expensive for small-scale dairy operations [252,268]. The economic feasibility of adopting such technologies is a crucial factor, especially for smaller producers with limited resources. The complexity of these technologies demands specialized knowledge and skilled personnel for operation and maintenance. Small dairy farms may lack the expertise required to effectively implement and manage these advanced systems [269]. Additionally, the need for continuous training and updating of skills can be a barrier for small-scale producers. These emerging technologies often require substantial initial investments in specialized equipment and infrastructure. The higher implementation costs compared to conventional processing technologies pose a significant barrier for adoption in resource-constrained settings [270]. Additionally, the industrial application of these novel technologies is still under development, which further increases costs and complexity [270]. Interestingly, while these technologies show clear environmental benefits by improving overall energy efficiency and reducing the use of nonrenewable resources [270], the lack of widespread adoption and economies of scale in developing countries may limit their cost-effectiveness. To alleviate this, crop residues, slurry, and other dairy-production residues could be utilized to enhance the sustainability of the dairy industry and make it more accessible to smaller farms [271,272]. The dairy industry generates various types of agricultural waste, including crop residues, animal manure, and food-processing waste. These residues, often considered waste, hold immense potential for improving sustainability and resource efficiency in dairy farming [273]. By recycling these residues into valuable resources, farmers can enhance soil fertility, reduce reliance on synthetic chemicals, and contribute to a more sustainable agricultural ecosystem [273]. While the utilization of agricultural waste can improve sustainability, there are some challenges to consider. For instance, legislation in some regions prohibits the application of untreated dairy manure directly to fields [271]. Additionally, the investments required for proper slurry storage and management may not always be proportional to the savings from lower nitrogen input and increased milk revenue, especially for smaller farms [274], making it, once again, difficult for smaller farms or developing countries. As such, the effective management and utilization of dairy-production residues can significantly contribute to the industry’s sustainability, although not without an initial investment. Practices such as composting, biogas production, and the use of slurry as organic fertilizer can help improve soil health, reduce environmental impacts, and potentially increase farm profitability [271,275]. For smaller farms, adopting these practices could lead to improved resource-use efficiency and reduced reliance on external inputs, making dairy farming more sustainable [276]. However, successful implementation requires proper knowledge dissemination, supportive legislation, and innovative technologies to overcome existing challenges [271,277]. The need for skilled personnel to operate and maintain these advanced production systems can be challenging in regions with limited technical expertise. While these technologies offer advantages such as improved microbial safety, preservation of nutritional compounds, and reduced processing times [262,278], their effects on the unique properties of small-animal dairy products are not yet fully understood. The scale of production in small-animal dairy farms may not justify the implementation of these technologies, which are often designed for larger-scale operations. Adapting these technologies to smaller production volumes while maintaining efficiency and cost-effectiveness can be challenging [252,279]. Considering this, while technologies like UV-C, ohmic heating, ultrasound processing, electric field treatments, and others show promise for improving dairy processing, their implementation in the small-animal dairy industry faces hurdles related to cost, expertise, product-specific effects, and scale of operation. Further research and development focused on addressing these challenges could help make these technologies more accessible and practical for small-scale dairy producers in the future [279].

## 6. Conclusions

Research on novel processing technologies is crucial for the advancement of the dairy industry, particularly for small-scale milk-producing animals. The industry faces not only technological challenges but also issues related to education, research, health management, and economic sustainability [280]. Investigating the production of milk from smaller dairy species, such as yaks, zebu, camels, and others, is becoming increasingly relevant due to various factors. These animals play essential roles in marginal and impoverished areas, contributing to food biodiversity and providing income for farmers [12]. Their unique characteristics, such as adaptability to specific environments, make them valuable alternatives to traditional dairy cows, especially in arid or semi-arid regions. Research on these species is expanding, focusing on improving production technologies, understanding milk composition, and developing new dairy products [12]. As the global demand for milk increases, these smaller dairy species offer potential solutions for sustainable milk production in challenging environments.

It is necessary to promote the development of this industry and improve its productivity, particularly in developing countries [281], where both goat and sheep milk significantly contribute to the available milk supply. Implementing animal health control plans, especially parasitic control, is critical to addressing the industry’s challenges [281]. Alternative approaches, such as botanical dewormers and improved nutrition, can help manage gastrointestinal parasites, which are major limitations to production [282]. Additionally, the use of alternative feeds, such as crop residues and agro-industrial by-products, can increase productivity, reduce carbon footprint, and improve the quality of both meat and milk [283]. These alternatives offer cost-effective and environmentally friendly solutions, addressing issues like anthelmintic resistance and the high cost of commercial treatments. By focusing on planned health management and sustainable practices, the small-ruminant industry can overcome challenges and contribute to global food production and economic development [280,281].

## Figures and Tables

**Table 1:** Reported concentration of molecular components in milk from different species. The information is presented in the units of measurement reported by the authors.

		Sheep	Goat	Donkey	Zebu	Yak	Buffalo	Camel	Reindeer	Llama	Alpaca
Protein	αs-Casein	29.5% total [165]	24.8% total [161]	1.4–2.0 g/L [166]	-	0.8–1.2 g/100 g [159]	-	2.4–3.9 g/L [158]	-	-	-
	βs-Casein	61.6% total [165]	54.8% total [161]	3.9 g/L [166]	-	1.7–2.3 g/100 g [159]	-	5.5–29 g/L [158]	-	-	-
	k-Casein	8.9% total [165]	20.4% total [161]	Traces [160]	-	0.4–0.7 g/100 g [159]	-	0.1–2.4 g/L [158]	-	-	-
	Total casein	4.46 ± 0.82 g/100 g [162]	24 g/kg [161]	6.6 g/L [160]	-	3.2–4.0 g/100 g [159]	-	-	-	-	-
	Total protein	5.83 ± 0.79 g/100 g [162]	37.2 g/kg [161]	13.28 g/L [160]	34.9–40.9 g/L [167]	4.5–5.5 g/100 g [159]	4 g/100 g milk [163]	3.5 g/100 g [159]	10.4 g/100 g [163]	4.1 g/100 g [163]	5.8 g/100 g [163]
Fat	Cholesterol	288.4 ± 42.2 mg/100 g fat [164]	341.8 ± 15.6 mg/100 g fat [164]	8.6 mg/100 g [80]	-	22 mg/100 g [159]	-	37.0 g/100 g [159]	-	-	-
	Total fat	7.28 ± 1.10 g/100 g [162]	3.5 g/100 mL [29]	1.21 g/100 g [160]	43 g/kg [167]	7.22 g/100 g [168]	7.4 g/100 g milk [163]	4.5 g/100 g [159]	16.1 g/100 g [163]	4.2 g/100 g [163]	3.2 g/100 g [163]
Lactose		4.8 ± 0.4 g/100 g [169]	4.1 ± 0.4 g/100 g [169]	6.23 g/100 g [160]	48 g/kg [167]	4.4 g/100 g [159]	4.4 g/100 g [163]	4.4 g/100 g [159]	2.9 g/100 g [163]	6.9% total [142]	6.99% total [142]
Non-protein nitrogen		0.20% [164]	0.40% [164]	-	-	0.04–0.05% (total N) [170]	-	-	-	-	-
α-Lactalbumin		13.5% total whey [165]	24% total whey [161]	1.8–3.0 g/L [166]	-	0.2–0.4 g/100 g [159]	-	0.3–2.9 g/L [158]	-	340 mg/100 g [171]	-
β-Lactoglobulin		45.7% total whey [165]	53% total whey [161]	3.2–3.7 g/L [166]	-	0.4–0.7 g/100 g [159]	-	-	-	-	-
Ash		0.93 ± 0.04 g/100 g [162]	0.8 ± 0.01 g/100 g [169]	0.43 g/L [160]	7.8 g/L [167]	0.79 ± 0.05 g/100 g [170]	0,8 g/100 g [163]	-	1.5 g/100 g [163]	0.7 g/100 g [163]	1.6 g/100 g [163]
Somatic cells/ml		1.7 × 10^6^ ± 1.01 cel/mL [162]	9.3 × 10^5^ cel/mL [172]	(8.1 ± 2.5) × 10^3^ cel/mL [173]	71–1296 cel/L [167]	-	-	-	-	93.9 cel/mL [142]	83.9 cel/mL [142]
Total solids		17.32 ± 1.82 g/100 g [162]	12.25 ± 1.94 g/100 g [174]	1.5–1.8 g/100 mL [173]	131.9–147.5 g/L [167]	16.88 ± 1.36 g/100 g [170]	-	12.8 g/100 g [159]	-	16.03% [142]	16.41% [142]

**Table 2:** Summary of emerging technologies tested for processing milk from non-traditional animals. The technologies applied to dairy products from each non-traditional animal species are indicated with a check mark (✓).

Emerging Technologies Tested for Milk from Non-Traditional Animals	Technology		Sheep	Goat	Donkey	Zebu	Yak	Buffalo	Camel	Reindeer	Llama	Alpaca
Non thermal technologies	High-pressure processing	HPP	✓ [181,182]	✓ [179]	✓ + T° [183]	-	✓ [184]	✓ [185]	✓ [186]	-	-	-
	Pulse electric field	PEF	-	✓ + T° [187]	-	-	-	-	-	-	-	-
	Ultrasound processing	USP	✓ [188]	✓ [189]	✓ [190]	-	-	✓ [191]	✓ [192]	-	-	-
	UV-C	UV	✓ + T° [193]	✓ [194]	✓ [195]	-	-	✓ [196]	✓ [197]	-	-	-
	Gamma radiation	GR	✓ [198]	✓ [199]	-	-	-	-	✓ [200]	-	-	-
	Microfiltration	MF	✓ [201]	✓ [202]	-	-	-	-	-	-	-	-
	Cold plasma processing	CPP	✓ [203]	✓ [204]	-	-	-	-	-	-	-	-
Thermal technologies	Ohmic heating	OH	✓ [205]	✓ [206]	-	-	-	✓ [207]	✓ [208]	-	-	-

## Data Availability

Not applicable.
